# CHIP regulates skeletal development and postnatal bone growth

**DOI:** 10.1002/jcp.29424

**Published:** 2020-01-03

**Authors:** Wenbo Wang, Jun Li, Frank C. Ko, Xia Zhao, Yusen Qiao, Ronald S. Lu, D. Rick Sumner, Tingyu Wang, Di Chen

**Affiliations:** ^1^ Department of Orthopedic Surgery Rush University Medical Center Chicago Illinois; ^2^ Department of Cell and Molecular Medicine Rush University Medical Center Chicago Illinois; ^3^ Department of Pharmacy, Shanghai Ninth People's Hospital Shanghai JiaoTong University School of Medicine Shanghai China

**Keywords:** bone formation, CHIP, conditional knockout, NF‐κB signaling, skeletal development

## Abstract

C terminus of Hsc70‐interacting protein (CHIP) is a chaperone‐dependent and U‐box containing E3 ubiquitin ligase. In previous studies, we found that CHIP regulates the stability of multiple tumor necrosis factor receptor‐associated factor proteins in bone cells. In *Chip* global knockout (KO) mice, nuclear factor‐κB signaling is activated, osteoclast formation is increased, osteoblast differentiation is inhibited, and bone mass is decreased in postnatal *Chip* KO mice. To determine the role of *Chip* in different cell types at different developmental stages, we created *Chip* ^*flox/flox*^ mice. We then generated *Chip* conditional KO mice *Chip* ^*CMV*^ and *Chip* ^*OsxER*^ and demonstrated defects in skeletal development and postnatal bone growth in *Chip* conditional KO mice. Our findings indicate that *Chip* conditional KO mice could serve as a critical reagent for further investigations of functions of CHIP in bone cells and in other cell types.

## Graphical abstract

In this study, we observed defects in skeletal development and postnatal bone growth in C terminus of Hsc70‐interacting protein (CHIP) conditional knockout mice.

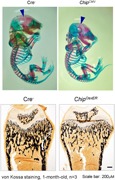

## 1 INTRODUCTION

Skeletal development and endochondral bone formation is a complicated process involving chondrocyte proliferation, differentiation and hypertrophy, cartilage calcification, resorption of calcified cartilage, vascular invasion, and osteoblast differentiation. All of these steps are regulated precisely by multiple growth factors and signaling molecules. Nuclear factor‐κB (NF‐κB) signaling controls osteoclast formation (Boyce, Yao, & Xing, 2010) and is involved in the process of removal of calcified cartilage. In NF‐κB p50/p65 double knockout (KO) mice, osteoclast formation is severely impaired leading to defects in the resorption of calcified cartilage and largely expanded hypertrophic zone at the postnatal stage (Xing, Chen, & Boyce, 2013).

NF‐κB signaling plays an important role in bone biology. C terminus of Hsc70‐interacting protein (CHIP) is a chaperone‐dependent and U‐box containing E3 ubiquitin ligase. It targets the degradation of proteins critical for multiple cellular functions and signaling pathways. In previous studies, we found that CHIP controls the degradation of multiple tumor necrosis factor receptor‐associated factor proteins and regulates NF‐κB signaling in both osteoclasts and osteoblasts and NF‐κB signaling is activated in *Chip* KO mice (Li et al., 2014; Wang et al., 2018). It is known that activation of NF‐κB signaling leads to stimulation of osteoclast formation and inhibition of osteoblast differentiation (Abu‐Amer, 2013; Boyce et al., 2010; Jimi et al., 2016; Otero, Chen, Zhang, & Abu‐Amer, 2012; Park et al., 2007; Swarnkar, Zhang, Mbalaviele, Long, & Abu‐Amer, 2014; Yao et al., 2014). However, the role of NF‐κB signaling in skeletal development remains unclear. In the present studies, we investigated changes in skeletal development in *Chip* conditional KO mice.

To study the role of CHIP in bone remodeling and in aging in adult mice and to determine the tissue‐specific effects of *Chip* in bone and cartilage and other organs in the body, we have generated *Chip* ^*flox/flox*^ mice. To demonstrate that *Chip* ^*flox/flox*^ mice have been properly developed, we then created *Chip* ^*CMV*^ and *Chip* ^*OsxER*^ conditional KO mice. We analyzed bone phenotype of *Chip* conditional KO mice and demonstrated that *Chip* ^*flox/flox*^ mice could be used as an important tool to investigate functions of *Chip* in multiple tissues at different developmental stages.

## 2 MATERIALS AND METHODS

### 2.1 *Chip* KO Mice

**Figure 1:**
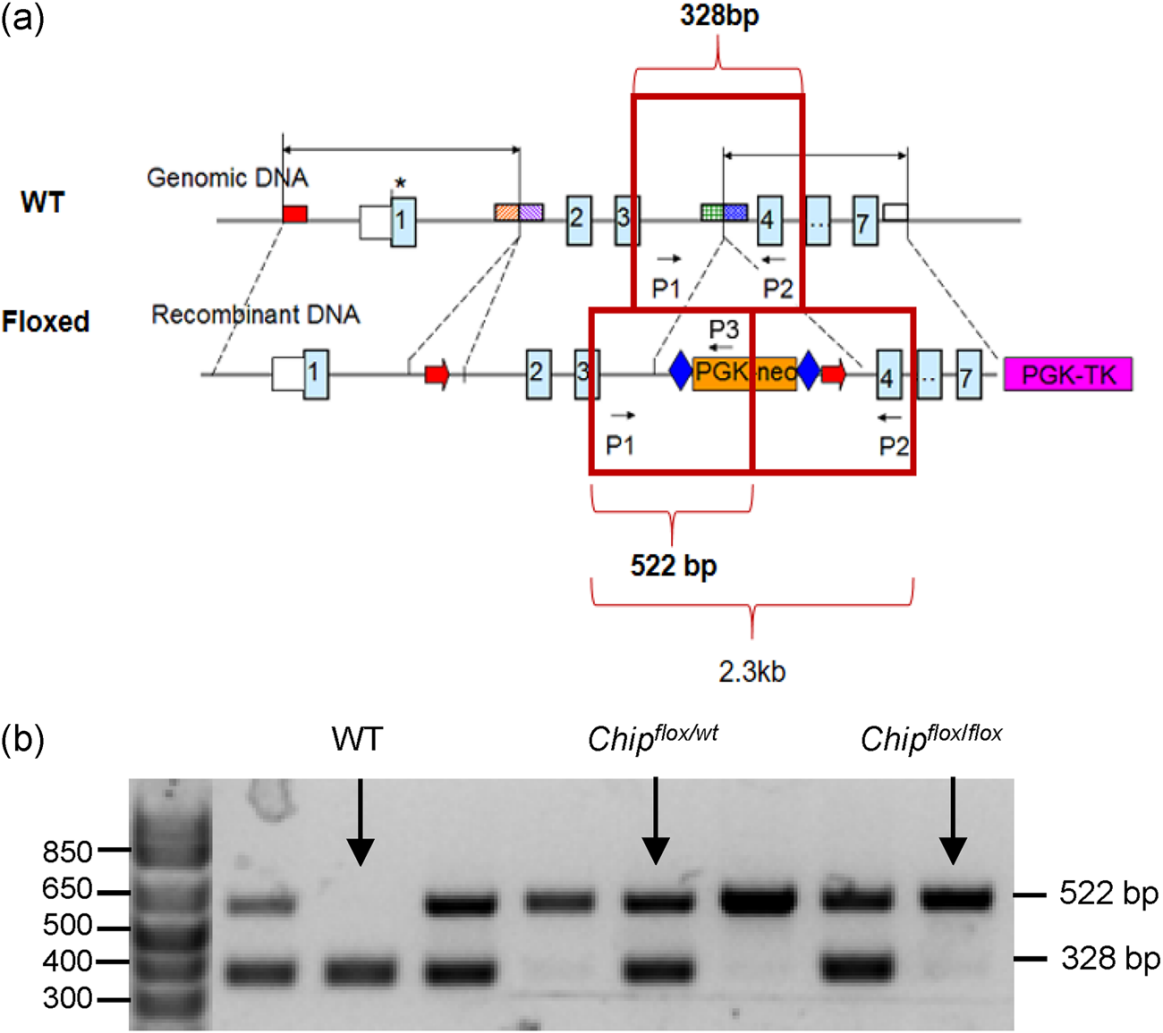
Genotyping *Chip‐flox* mice. *Chip‐flox* mice were genotyped with specific primers P1 (forward primer): 5′‐CATATCTCACCAGGCTCAT‐3′, P2 (reverse primer): 5′‐ACACACAATGACCCACAT‐3′, and P3 (reverse primer): 5′‐GGCCTACCCGCTTCCATTGCTC‐3′. Primer set P1/P3 amplifies a 522‐bp band to detect *Chip* ^*flox/flox*^ allele and primer set P1/P2 amplifies a 328‐bp band to detect WT allele. Both 328‐ and 522‐bp bands were detected in *Chip* ^*flox/wt*^ mice. WT, wild‐type

*Chip* ^*flox/flox*^ mice were generated in Nanjing Biomedical Research Institute (Nanjing University, Nanjing, China). In these mice the *Chip* gene was floxed at the flanking sites of exon 1 and exon 3. The primer sequences for genotyping *Chip* ^*flox/flox*^ mice were: P1 (forward primer): 5′‐CATATCTCACCAGGCTCAT‐3′, P2 (reverse primer): 5′‐ACACACAATGACCCACAT‐3′ and P3 (reverse primer): 5′‐GGCCTACCCGCTTCCATTGCTC‐3′. The genotyping result was presented in Figure 1. *CMV‐Cre* and *Osx‐CreER* transgenic mice were obtained from the Jackson Laboratory (Bar Harbor, Maine).

### 2.2 Whole embryo alizarin red/alcian blue staining

Embryos at E14.5 and E18.5 were collected and the skin, viscera, and adipose tissues were carefully removed. Whole skeletons were fixed in 95% ethanol for 2 days followed by fixation in acetone for an additional day, and stained with 0.015% alcian blue and 0.005% alizarin red for 3 days. Images of the skeletons were taken when most of the soft tissue was digested in 1% potassium chloride.

### 2.3 Microcomputed tomography analysis

We used a Scanco microcomputed tomography 35 scanner (Scanco Medical, Brüttisellen, Switzerland) with 55 kVp source and 145 μA current for formalin‐fixed mouse legs with a resolution of 10 μm. The scanned images from each group were evaluated at the same thresholds to allow three‐dimensional structural rendering of each sample.

### 2.4 Histology

Tibiae were harvested and fixed in 10% neutral‐buffered formalin for 3 days and decalcified for 14 days in 14% EDTA and then paraffin‐embedded. Three‐micrometer sections were cut and alcian blue/H&E orange G staining and tartrate‐resistant acid phosphatase (TRAP) staining were performed (Shu et al., 2013; B. Wang et al., 2014; Wang et al., 2017).

### 2.5 Statistical analysis

Data are presented as the mean ± standard deviation. For experiments comparing two groups of data, unpaired Student's *t* test was performed. A value of *p* < .05 was considered to be significant.

## 3 RESULTS

### 3.1 Skeletal development was delayed in *Chip* ^*CMV*^ conditional KO embryos

**Figure 2:**
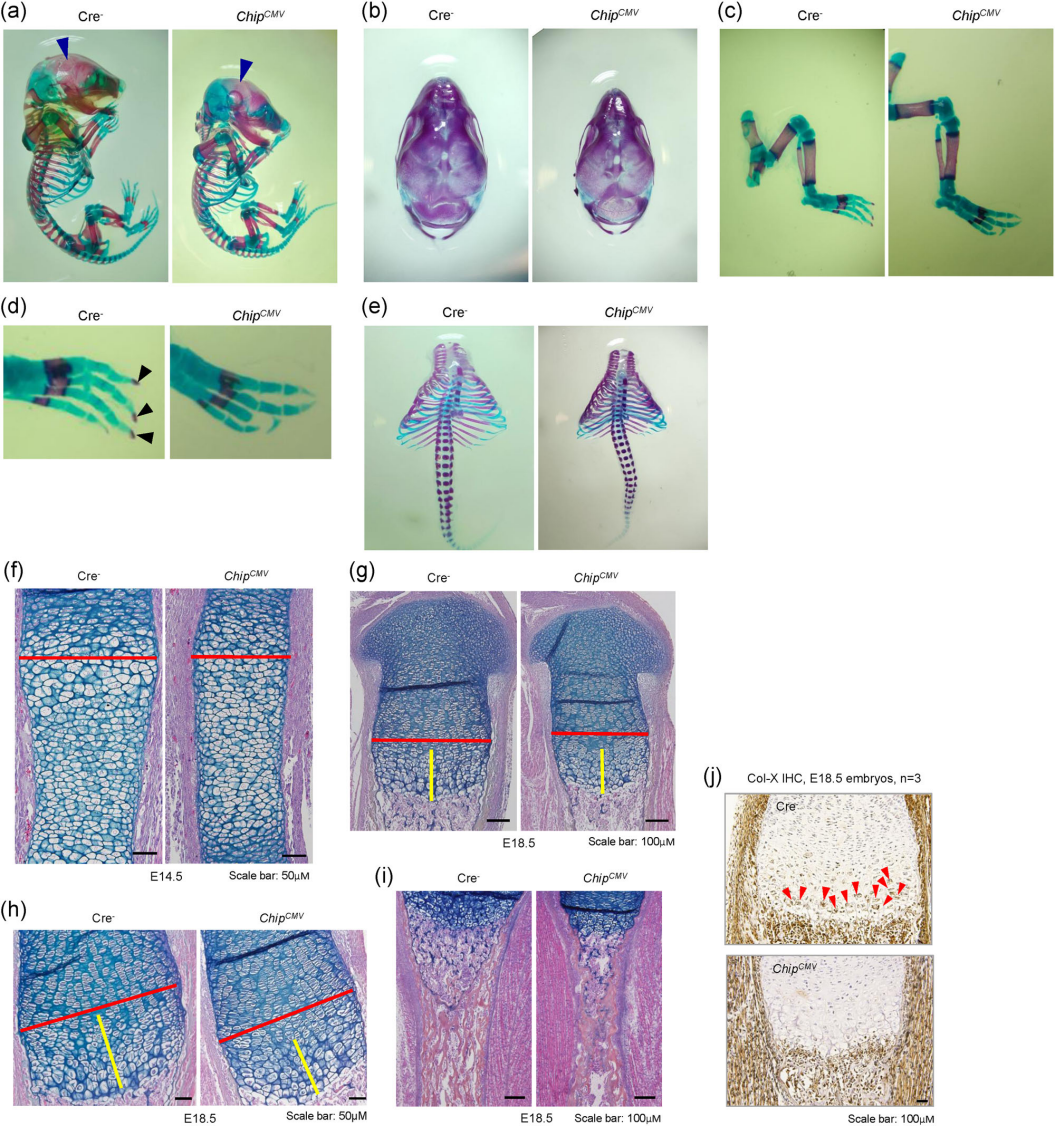
Skeletal development was delayed in *Chip* KO embryos. (a–e) *Chip* ^*flox/flox*^ mice were bred with *CMV‐Cre* mice and E18.5 *Chip* ^*CMV*^ KO embryos and Cre‐negative littermate embryos were analyzed by whole embryo alizarin red/alcian blue staining. (a) The size of *Chip* ^*CMV*^ KO embryo was smaller. The mineralization of the supraoccipital bone (a, blue arrowheads) and the digits (d, black arrowheads) was delayed in *Chip* ^*CMV*^ KO embryos. (f–i) *Chip* ^*flox/flox*^ mice were bred with *CMV‐Cre* mice and E14.5 and E18.5 *Chip* ^*CMV*^ KO embryos and Cre‐negative littermate embryos were analyzed by histology. (f) Analysis of E14.5 embryos showed that the size and width (red line) of the skeletal were significantly reduced in *Chip* ^*CMV*^ KO embryos. (g and h) Analysis of E18.5 embryos showed that the width (red lines) of the skeletal and the length of hypertrophic zone (yellow lines) were reduced in *Chip* ^*CMV*^ KO embryos. (i) The size of primary ossification center was smaller in E18.5 *Chip* ^*CMV*^ KO embryos. (j) Results of IHC staining showed that ColX expression (red arrowheads) was decreased in E18.5 *Chip* ^*CMV*^ KO embryos. IHC, immunohistochemistry; KO, knockout

In previous studies, we investigated the role of CHIP in postnatal bone growth. To determine the role of CHIP in skeletal development, in the present studies, we generated *Chip* ^*flox/flox*^ mice and *Chip* ^*CMV*^ conditional KO mice and analyzed changes in skeletal development in E14.5 and E18.5 *Chip* ^*CMV*^ KO embryos and Cre‐negative littermate embryos. Results of whole embryo alizarin red/alcian blue staining showed that sizes of *Chip* ^*CMV*^ KO embryos were smaller (Figure 2a). We also found that the mineralization in the supraoccipital bone and the distal phalanx of the limb were delayed in *Chip* ^*CMV*^ KO embryos (Figure 2b–d). Axial bone seems developed normally in *Chip* ^*CMV*^ KO mice (Figure 2e). Results of histological analysis showed that the width of limb of E14.5 embryos was reduced in *Chip* ^*CMV*^ KO embryos (Figure 2f) and the width and the length of the hypertrophic zones were also decreased in *Chip* ^*CMV*^ KO embryos (Figure 2g,h). The size of primary ossification center in the tibiae was smaller in *Chip* ^*CMV*^ KO embryos (Figure 2i). In addition, we also found that ColX expression was reduced in the hypertrophic zone of E18.5 *Chip* ^*CMV*^ KO embryos (Figure 2j).

### 3.2 Bone mass was decreased in *Chip* ^*CMV*^ conditional KO mice

**Figure 3:**
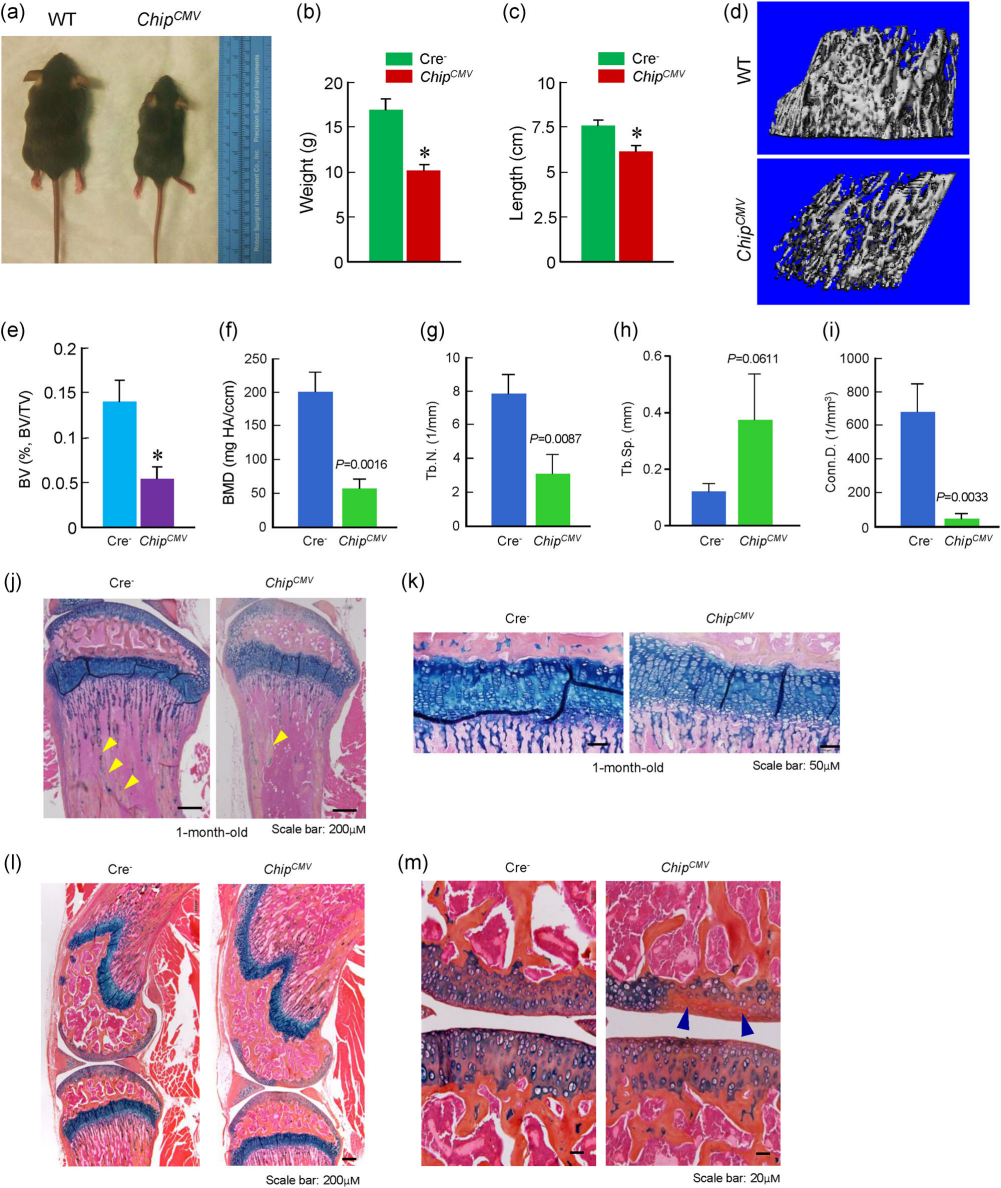
Bone mass was decreased in *Chip* ^*CMV*^ conditional KO mice. *Chip* ^*flox/flox*^ mice were bred with *CMV‐Cre* mice and *Chip* ^*CMV*^ conditional KO mice and Cre‐negative control mice were killed at 4 weeks of age. (a–c) The size, body weight, and body length of *Chip* ^*CMV*^ KO mice were significantly smaller than Cre‐negative controls. (d–i) Changes in bone mass were analyzed by µCT. (d) Bone mass, (e) bone volume, (f) bone mineral density (BMD), (g) trabecular number (Tb.N.), and (h) In contrast, trabecular separation (Tb.Sp.) was increased (did not reach the statistical significant) in *Chip^CMV^* KO mice. (i) connectivity density (Conn.D.) was significantly reduced in *Chip^CMV^* KO mice. (j–m) Changes in bone morphology were also analyzed by histology. (j) Bone mass was significantly decreased in *Chip* ^*CMV*^ KO mice (trabecular bone was indicated by yellow arrowheads). (k) No significant changes in the length of growth plate cartilage in *Chip* ^*CMV*^ KO mice. (l and m) In addition, histological analysis showed that the loss of articular chondrocytes and reduced alcian blue staining (blue arrowheads) was observed in *Chip* ^*CMV*^ KO mice. BV/TV, bone volume/total volume; KO, knockout; µCT, microcomputed tomography; WT, wild‐type

Our long‐term goal is to understand the specific roles of CHIP in bone remodeling and in aging in adult mice. To achieve this goal, we generated *Chip* ^*flox/flox*^ mice. We have bred *Chip* ^*flox/flox*^ mice with *CMV‐Cre* transgenic mice to determine if *Chip* ^*CMV*^ conditional KO mice mimic bone loss phenotype that we have observed in *Chip* ^*−/*−^ global KO mice. We analyzed 4‐week‐old *Chip* ^*CMV*^ KO mice and found that body weight and body length of *Chip* ^*CMV*^ KO mice were significantly reduced compared to the Cre‐negative littermates (Figure 3a–c). The μCT analysis showed that bone volume (bone volume/total volume, %) and bone mineral density (BMD) were significantly decreased in *Chip* ^*CMV*^ KO mice compared to the Cre‐negative littermates (Figure 3d–f). Consistent with the findings of bone mass reduction, the trabecular number was decreased and trabecular separation was increased in *Chip* ^*CMV*^ KO mice (Figure 3g,h). In contrast, the connectivity density was significantly decreased in *Chip* ^*CMV*^ KO mice (Figure 3i), suggesting that the bone quality were also reduced in *Chip* ^*CMV*^ KO mice. We then performed histological analysis and observed significant bone loss phenotype in *Chip* ^*CMV*^ KO mice (Figure 3j). In contrast, no significant changes in growth plate cartilage were found in *Chip* ^*CMV*^ KO mice (Figure 3k). In addition to the changes in bone mass, we also found defects in articular cartilage in *Chip* ^*CMV*^ KO mice, including loss of articular chondrocytes and reduced Alcian blue staining (Figure 3l,m), reflecting the loss of proteoglycan in articular cartilage in postnatal *Chip* ^*CMV*^ KO mice.

### 3.3 Bone mass was decreased in *Chip* ^*OsxER*^ conditional KO mice

**Figure 4:**
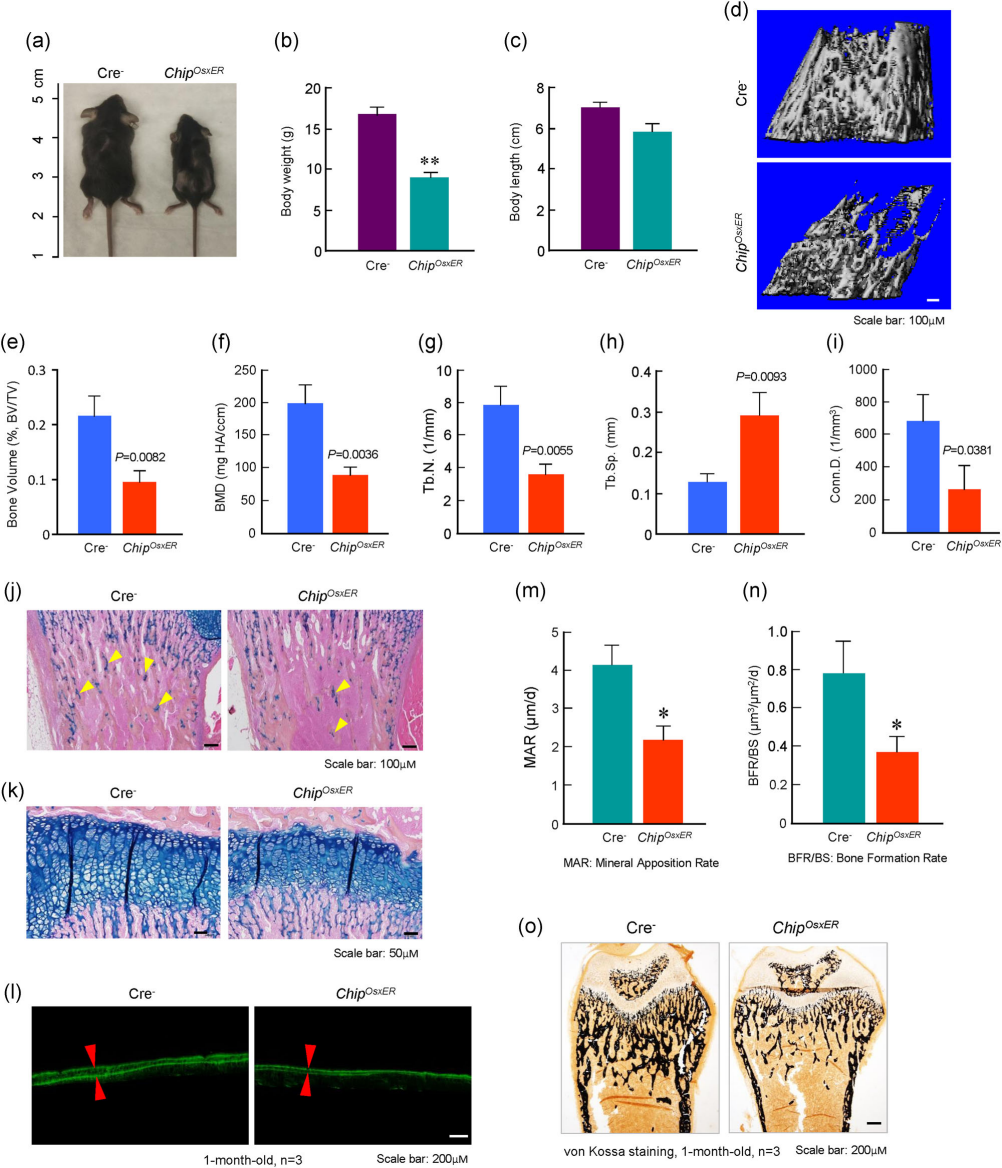
Bone mass was decreased in *Chip* ^*OsxER*^ conditional KO mice. *Chip* ^*flox/flox*^ mice were bred with *Osx‐CreER* transgenic mice and resultant *Chip* ^*OsxER*^ mice were administered with tamoxifen (1 mg/10 g body weight, i.p. injection, x 5 days) in 2‐week‐old *Chip* ^*flox/flox*^ (Cre‐negative control) mice and *Chip* ^*OsxER*^ mice. *Chip* ^*OsxER*^ KO mice were then killed at 4 weeks of age. (a and b) Changes in body size and body weight were observed in *Chip* ^*OsxER*^ KO mice. (c) In contrast, the body length was not significantly changed. (d–i) Bone mass was analyzed by µCT. The μCT images of bone structure and bone morphology showed reduced (d) bone mass, (e) bone volume, (f) bone mineral density (BMD), (g) trabecular number (Tb.N.), and (i) connectivity density (Conn.D.) were significantly reduced in *Chip* ^*OsxER*^ KO mice. (h) In contrast, trabecular separation (Tb.Sp.) was significantly increased in *Chip* ^*OsxER*^ KO mice. (j and k) Consistent with µCT analysis, histological results showed bone mass decrease in *Chip* ^*OsxER*^ KO mice (trabecular bone is indicated by yellow arrowheads). In contrast, no obvious changes in growth plate cartilage morphology were observed in *Chip* ^*OsxER*^ KO mice. (l–n) We also performed calcein/calcein double labeling assay and results showed that mineral apposition rates (MAR) and bone formation rates (BFR) were significantly reduced *Chip* ^*OsxER*^ KO mice. (o) Results of von Kossa staining showed that the mineralized bone formation was also reduced in *Chip* ^*OsxER*^ KO mice. BV/TV, bone volume/total volume; KO, knockout; µCT, microcomputed tomography

In previous studies, we found bone loss phenotype in *Chip* global KO mice. However, it is not known if CHIP exerts its effect directly through bone cells or CHIP acts on bone through an indirect mechanism. To determine the specific effect of *Chip* on postnatal bone growth, we generated *Chip* ^*OxsER*^ conditional KO mice by breeding *Chip* ^*flox/flox*^ mice with *Osx‐CreER* transgenic mice, which target osteoblast precursor cells (Maes et al., 2010). Tamoxifen was administered into 2‐week‐old *Chip* ^*OxsER*^ mice and Cre‐negative littermates (*Chip* ^*flox/flox*^ mice). The results of a significant decrease in body weight and slightly reduced body size were found in *Chip^OxsER^* KO mice (Figure 4a–c). Results of μCT analysis showed that bone volume, BMD, trabecular numbers, and connectivity density were significantly reduced and trabecular separation was significantly increased in *Chip* ^*OxsER*^ KO mice (Figure 4d–i). We also analyzed bone morphological changes with histological method in 4‐week‐old *Chip* ^*OxsER*^ KO mice. Trabecular bone loss phenotype was found in *Chip* ^*OxsER*^ KO mice (Figure 4j). These findings suggest that bone mass and bone quality were reduced in *Chip* ^*OsxER*^ KO mice and indicate that local produced CHIP could directly regulate bone mass. In contrast, we did not observe significant changes in growth plate cartilage (Figure 4k). To further determine changes in bone formation in *Chip* ^*OsxER*^ KO mice, we performed calcein/calcein double labeling assay. We found that mineral apposition rates and bone formation rates were significantly decreased in *Chip* ^*OsxER*^ KO mice (Figure 4l–n). Consistent with these findings, results of von Kossa staining showed that mineralized trabecular bone volume was significantly reduced in *Chip* ^*OsxER*^ KO mice (Figure 4o).

**Figure 5:**
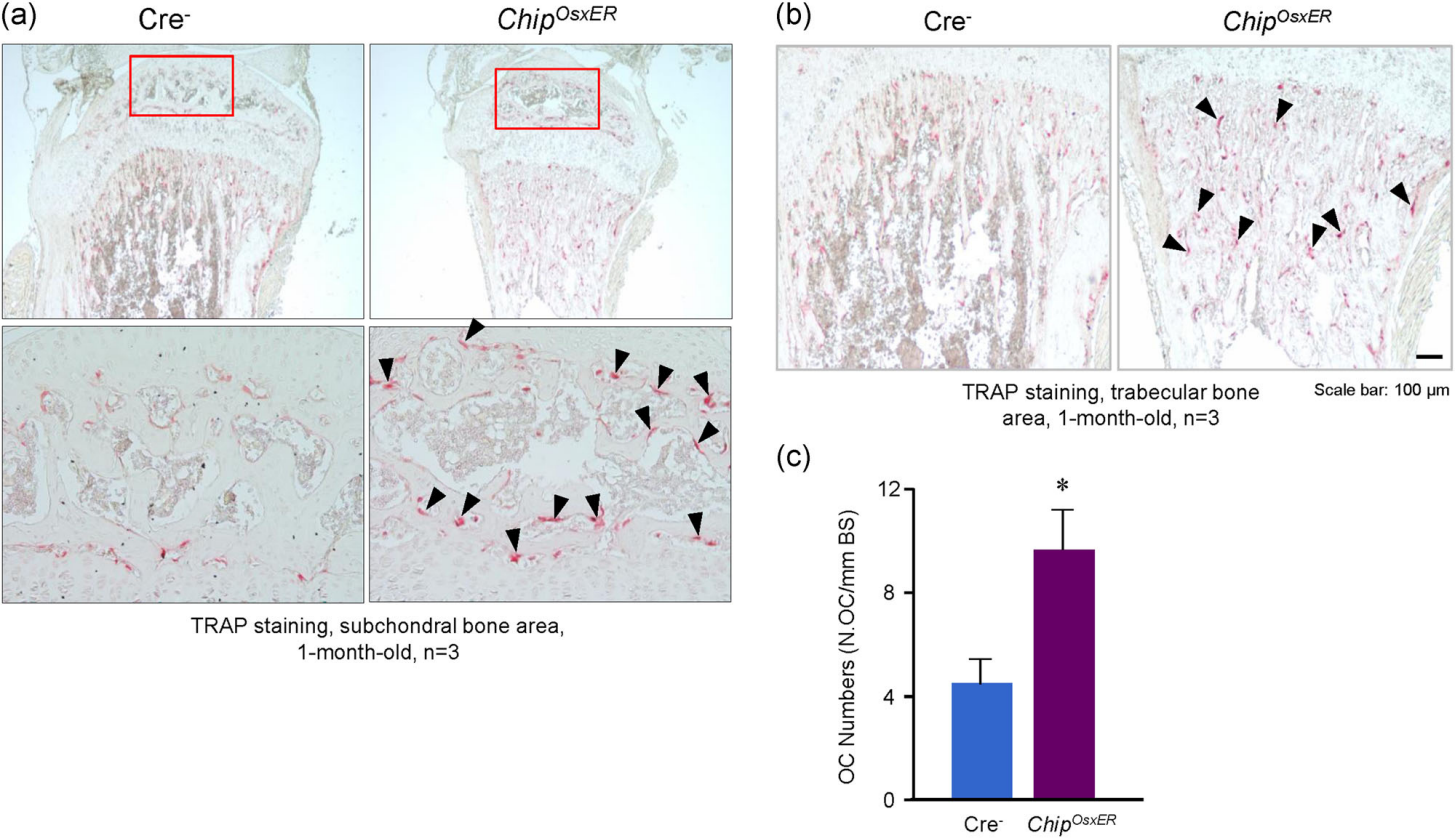
TRAP‐positive osteoclast formation was increased in *Chip* ^*OsxER*^ conditional KO mice. *Chip* ^*flox/flox*^ mice were bred with *Osx‐CreER* transgenic mice and resultant *Chip* ^*OsxER*^ KO mice were administered with tamoxifen in 2‐week‐old *Chip* ^*flox/flox*^ (Cre‐negative control) mice and *Chip* ^*OsxER*^ mice and the mice were killed at 4 weeks of age. TRAP staining was performed. Significant increases in the numbers of TRAP‐positive cells were detected in subchondral bone areas (a) and in trabecular bone areas underneath the growth plate (b and c) in *Chip* ^*OsxER*^ KO mice. KO, knockout; TRAP, tartrate‐resistant acid phosphatase

In previous studies, we demonstrated that NF‐κB signaling is activated in *Chip* global KO mice (Wang et al., 2018). In this study, we performed TRAP staining in subchondral bone areas and in trabecular bone areas underneath the growth plate. We found that osteoclast formation was significantly increased in *Chip* ^*OxsER*^ KO mice (Figure 5a–c), suggesting that osteoclast formation was also affected by *Chip* ^*OxsER*^ KO mice.

## 4 DISCUSSION

In previous studies, we demonstrated that CHIP controls NF‐κB signaling and regulates postnatal bone growth. In *Chip* global KO mice, osteoclast formation and bone resorption were increased and osteoblast differentiation was decreased (Li et al., 2014; Wang et al., 2018). In the present studies, we generated *Chip* ^*flox/flox*^ mice and then created *Chip* ^*CMV*^ and *Chip* ^*OsxER*^ conditional KO mice. We analyzed changes in skeletal development in *Chip* ^*CMV*^ KO embryos and we also analyzed changes in postnatal bone growth and bone mass in 1‐month‐old *Chip* ^*CMV*^ and *Chip* ^*OsxER*^ conditional KO mice. Our findings suggest that CHIP plays an important role in skeletal development and postnatal bone growth and regulates bone mass. Our findings indicate that we have successfully generated *Chip* conditional KO mice and confirmed that *Chip* is expressed in *Osx*‐expressing osteoblast precursor cells and plays a specific role in the regulation of bone mass.

Our previous study demonstrated that CHIP regulates NF‐κB signaling through inducing the degradation of multiple TRAF proteins, including TRAF2, TRAF5, and TRAF6 (Li et al., 2014; Wang et al., 2018). It is known that NF‐κB controls growth plate cartilage development. In NF‐κB p50/p65 double KO mice, osteoclast formation is severely impaired leading to defects in the resorption of calcified cartilage and largely expanded hypertrophic cartilage zone at the postnatal stage (Xing et al., 2013). In this study, we found that growth plate cartilage development relatively normal in postnatal *Chip* ^*CMV*^ and *Chip* ^*OsxER*^ KO mice. These findings suggest that the activation of NF‐κB signaling may not be able to significantly affect postnatal growth plate cartilage development although osteoclast formation is increased in *Chip* KO mice.

Bone is an endocrine organ and bone remodeling is a dynamic process that is active throughout the entire life. To determine the specific role of CHIP at different cell populations and at the different developmental stages of life, it requires the generation of inducible *Chip* conditional KO mice. As a long‐term goal of this project, we will determine the roles of *Chip* in osteoclast and osteoblast lineage cells at different developmental stages. We have recently generated *Chip* ^*flox/flox*^ mice and *Chip* conditional KO mice which allow us to perform tissue‐specific and longitudinal studies to investigate the role of CHIP in bone remodeling.

CHIP may play an important role in aging. Sirtuin 6 (SirT6) is a stress‐responsive protein deacetylase and mono‐ADP ribosyltransferase enzyme encoded by the *SirT6* gene (Min et al., 2008). Studies in mice have revealed that *Sirt6* is essential for postnatal development and survival. SirT6 functions in multiple molecular pathways related to aging, including DNA repair, telomere maintenance, glycolysis, and inflammation (Frye, 2000). SirT6 promotes resistance to DNA damage and oxidative stress, the defects closely related to age‐associated diseases (Beauharmois, Bolivar, & Welch, 2013). CHIP prevents proteasome‐dependent degradation of SirT6 and SirT6 stability is increased in *Chip*‐deficient cells. These results suggest that CHIP protects proteasomal degradation of SirT6. To study the roles of CHIP in SirT6 and other proteins associated with aging, we need to create *Chip* ^*flox/flox*^ mice and *Chip* conditional KO mice since *Chip* global KO mice died at postnatal or early adult stages. We have recently generated *Chip* ^*CMV*^ and *Chip* ^*OsxER*^ conditional KO mice, which showed bone loss phenotype as we observed in *Chip* global KO mice (Li et al., 2014; Wang et al., 2018), suggesting that *Chip* ^*flox/flox*^ mice could be used for long‐term and longitudinal studies.

The roles of CHIP in physiological functions and disease initiation and progression have been extensively investigated in recent years. For example, CHIP plays a critical role in neurodegenerative diseases, inflammation, and cardiovascular diseases. Generation and making the availability of *Chip* ^*flox/flox*^ mice to other fields will make studies of CHIP functions in other organ systems easier.

## CONFLICT OF INTERESTS

The authors declare that there are no conflict of interests.

## AUTHOR CONTRIBUTIONS

W. W., J. L., F. C. K., X. Z., Y. Q., and R. S. L. carried out experiments. T. W. and D. C. prepared the manuscript, contributed to the experimental design, data interpretation, and finalized the manuscript. D. R. S. revised the manuscript.

## ETHICS STATEMENT

The animal protocol of this study has been approved by the IACUC of the Rush University Medical Center and all experimental methods and procedures were carried out in accordance with the approved guidelines.

## Data Availability

The data that support the findings of this study are available from the corresponding author upon reasonable request.
